# Mild hypothermia alleviates brain oedema and blood-brain barrier disruption by attenuating tight junction and adherens junction breakdown in a swine model of cardiopulmonary resuscitation

**DOI:** 10.1371/journal.pone.0174596

**Published:** 2017-03-29

**Authors:** Jiebin Li, Chunsheng Li, Wei Yuan, Junyuan Wu, Jie Li, Zhenhua Li, Yongzhen Zhao

**Affiliations:** 1 Beijing Key Laboratory of Cardiopulmonary-Cerebral Resuscitation, Department of Emergency Medicine, Beijing Chaoyang Hospital, Capital Medical University, Beijing, China; 2 Department of Emergency Medicine, Beijing FuXing Hospital, Capital Medical University, Beijing, China; 3 Department of Emergency Medicine, Beijing Friendship Hospital, Capital Medical University, Beijing, China; Hungarian Academy of Sciences, HUNGARY

## Abstract

Mild hypothermia improves survival and neurological recovery after cardiac arrest (CA) and cardiopulmonary resuscitation (CPR). However, the mechanism underlying this phenomenon is not fully elucidated. The aim of this study was to determine whether mild hypothermia alleviates early blood–brain barrier (BBB) disruption. We investigated the effects of mild hypothermia on neurologic outcome, survival rate, brain water content, BBB permeability and changes in tight junctions (TJs) and adherens junctions (AJs) after CA and CPR. Pigs were subjected to 8 min of untreated ventricular fibrillation followed by CPR. Mild hypothermia (33°C) was intravascularly induced and maintained at this temperature for 12 h, followed by active rewarming. Mild hypothermia significantly reduced cortical water content, decreased BBB permeability and attenuated TJ ultrastructural and basement membrane breakdown in brain cortical microvessels. Mild hypothermia also attenuated the CPR-induced decreases in TJ (occludin, claudin-5, ZO-1) and AJ (VE-cadherin) protein and mRNA expression. Furthermore, mild hypothermia decreased the CA- and CPR-induced increases in matrix metalloproteinase-9 (MMP-9) and vascular endothelial growth factor (VEGF) expression and increased angiogenin-1 (Ang-1) expression. Our findings suggest that mild hypothermia attenuates the CA- and resuscitation-induced early brain oedema and BBB disruption, and this improvement might be at least partially associated with attenuation of the breakdown of TJ and AJ, suppression of MMP-9 and VEGF expression, and upregulation of Ang-1 expression.

## Introduction

Brain injury remains a significant source of morbidity in cardiac arrest (CA) and cardiopulmonary resuscitation (CPR) survivors. One of the most serious complications of brain injury following CA is brain oedema [1,2], which is associated with adverse neurologic sequelae and death [2,3].

Mild hypothermia management has been shown to exert a variety of neuroprotective effects in CA patients and animals [4–7]. These protective effects may result from inhibition of inflammatory cytokines, brain metabolism and oxidative stress, reduction of mitochondrial membrane permeability, attenuation of neuronal apoptosis, amelioration of brain oedema and other processes [1,3,5–8]. However, the effects of mild hypothermia on CA- and resuscitation-induced blood-brain barrier (BBB) disruption have not been fully elucidated.

The BBB is responsible for regulating and limiting the movement of plasma constituents into the brain parenchyma [9,10], and disruption of the BBB is a major factor leading to permanent cerebral oedema after CA and resuscitation [11–14]. The BBB comprises cerebral microvascular endothelial cells, a capillary basement membrane, pericytes and astrocyte endfeet [9,15,16]. The junctional complexes between endothelial cells include tight junctions (TJs) and adherens junctions (AJs) [15]. TJs are the main structural and functional molecules responsible for preserving the integrity of the microvascular endothelium in the brain [9]. TJs are composed of occludin, claudin, and junction adhesion molecules as well as many cytoplasmic accessory proteins, including ZO-1 and cingulin [16–18]. AJs play a role in maintaining the structural stability of TJs and are essentially composed of vascular endothelial (VE)-cadherin [9,17]. Matrix metalloproteinase-9 (MMP-9), vascular endothelial growth factor (VEGF) and angiogenin-1 (Ang-1) perform important functions in maintaining TJ and AJ integrity [16,17,19–23]. Ischaemic injury decreases TJ integrity between endothelial cells in the brain, resulting in BBB breakdown and directly contributing to brain vasogenic oedema, haemorrhagic transformation and increased mortality [17,24].

The aim of this study was to determine the effects of whole-body mild hypothermia on BBB breakdown in our pig model of CA and resuscitation. We tested the hypothesis that mild hypothermia alleviates disruption of the aforementioned TJ-related proteins occludin, claudin-5, and ZO-1 and the AJ-related protein VE-cadherin in cerebral cortical tissue. We also examined whether changes in MMP-9, VEGF, and Ang-1 expression were associated with these effects.

## Materials and methods

### Animal preparation

Thirty-four male healthy Beijing Landrace pigs (license number SCXK 11-00-002, the Registered Laboratory Animal Center in Beijing, China) aged 12–14 weeks and weighing 30±2.3 kg were used for this study. All animals were fasted preoperatively overnight and were allowed free access to water. This study was conducted in strict accordance with recommendations in the Guide for the Care and Use of Laboratory Animals of the Capital Medical University Animal Care and Use Committee. This project was approved by the Committee on the Ethics of Animal Experiments of Capital Medical University (permit number 2010-D-013), China. All surgeries were performed under anaesthesia and analgesia, and all efforts were made to minimize suffering. The reporting of this study complies with the ARRIVE guidelines.

The pigs were sedated with 0.5 mg/kg intramuscular midazolam, followed by ear vein injection of propofol (1.0 to 2.0 mg/kg). To maintain all animals under anaesthesia and unconscious, anaesthesia and analgesia were maintained intravenously via continuous infusion of 3% sodium pentobarbital (8 mg/kg/h) and fentanyl (5 μg/kg/h) after tracheal intubation. Anaesthesia was maintained continuously using pentobarbital until 18 hours after ROSC, followed by intravenous injection of propofol (0.4 mg/kg) when necessary. We used propofol before assessing pig neurologic outcome since recovery from propofol anaesthesia is more rapid and complete than recovery from sodium pentobarbital anaesthesia.

Ventilation was modulated with a volume-controlled ventilator (Evata IV, Drager Medical, Lubeck, Germany) administering room air at a ventilation rate of 12 to 20 breaths/minute, with a tidal volume of 8 to 15 mL/kg and a positive end-expiratory pressure of 5 mmHg. End-tidal PCO_2_ was measured continuously by a capnometer (NPB-75; Nellcor Puritan Bennett, Inc., Pleasanton, CA). The respiratory rate and tidal volume were adjusted to maintain the end-tidal PCO_2_ between 35 and 45 mmHg. Arterial blood gases (GEM Premier 3000 Blood Gas Analyzer, Instrumentation Laboratory, Lexington, MA) were analysed to guide adjustment of the above ventilation parameters. Standard lead II electrocardiograms, capnograms, and oxygen saturation were monitored with a patient monitoring system (M1165; Hewlett-Packard, Palo Alto, CA, USA). A 7F central venous catheter was inserted in the left internal jugular vein to measure central venous pressure (CVP) and to inject ice-cold saline. To measure aortic blood pressure and heart rate, a 4F thermistor-tipped catheter for arterial thermodilution (Pulsion Medical Systems, Munich, Germany) was inserted into the right femoral artery. These arterial and central venous catheters were connected to an integrated bedside monitor (PICCO; Pulsion Medical Systems, Munich, Germany) for continuous haemodynamic monitoring. Discontinuous measurements of cardiac output were performed by injecting 10 mL of ice-cold saline into the central venous catheter. A 7F sheathing canal (Edwards Life Sciences, Irvine, CA) was inserted into the right internal jugular vein to place a temporary pacemaker conductor for induction of ventricular fibrillation (VF), after which the catheter was removed. A central venous cooling catheter (Icy, Alsius Corp., Irvine, CA, USA) was inserted into the inferior vena cava via the left femoral vein to induce hypothermia, and a temperature-sensing Foley catheter (Integral Medical Products Shaoxing, China) was inserted into the bladder after fistulation to measure core body temperature. All catheters were flushed and filled with heparinized normal saline (5 U/mL) to prevent clotting. The pigs were transfused with acetated Ringer’s solution (2 to 10 mL/kg/h intravenously) to maintain a CVP of 5 to 12 mmHg. Room temperature was adjusted to 26°C, and intra-bladder temperature was maintained at 38°C under an electrical fan and ice bags or a heating lamp and warm packs.

### Experimental protocol

**Fig 1:**
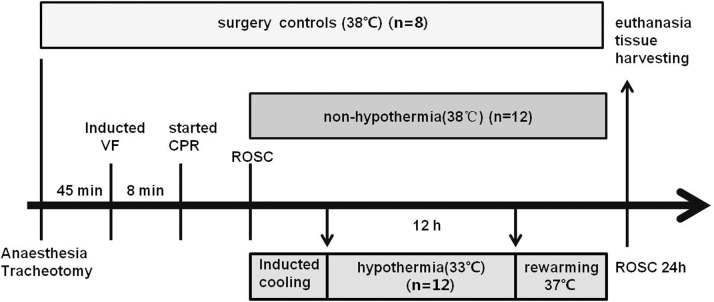
Experimental procedure. VF, ventricular fibrillation; CPR, cardiopulmonary resuscitation; ROSC, restoration of spontaneous circulation.

After preparation, 45 minutes were allotted for haemodynamic stabilization. The experimental procedure is presented in Fig 1. The temporary pacemaker conductor was inserted into the right ventricle, and VF was subsequently induced using a programmed electrical stimulation instrument (GY-600A; Kaifeng Huanan Instrument Ltd., Kaifeng, Henan, China) in S1/S2 mode (300/200 ms, 8:1 ratio, 40 V, and 10-ms step length) [25]. VF was confirmed via identification of its characteristic electrocardiographic waveform and observation of an abrupt decrease in mean arterial pressure (MAP) to 15 to 20 mmHg. Mechanical ventilation was discontinued after VF onset. After being subjected to 8 minutes of untreated VF, the pigs were ventilated with room air using a bag respirator. CPR was performed by two well-trained experimenters for 2 min at a compression rate of 100±5 beats per minute, a compression depth of 50±1 mm and a compression-to-ventilation ratio of 30:2. Chest compression quality was monitored using a Heartstart MRx Monitor/Defibrillator (M3535A; Philips Medical Systems, Best, Holland). External defibrillation (biphasic, 150 J; Philips Medical Systems, Andover, MA, USA) was attempted after 2 min of CPR. If VF persisted, CPR was resumed for 2 min. Then, 200-J shocks were applied in all subsequent defibrillation attempts. The animals were pronounced dead if spontaneous circulation was not restored within 30 minutes [25]. Return of spontaneous circulation (ROSC) was defined as persistence of a systolic blood pressure greater than 60 mmHg for at least 10 minutes, in accordance with the Utstein-style guidelines [6,26]. Successfully resuscitated animals were mechanically ventilated with 50% inspired oxygen for the first 30 minutes after resuscitation and with 21% inspired oxygen thereafter.

Eight pigs not subjected to VF or mild hypothermia served as the surgery control group (SC, n = 8). These pigs underwent the same operation and were subjected to the same intensive care protocol as the other pigs. Twenty-six pigs were subjected to VF and CPR. ROSC was not achieved in two animals, which were pronounced dead. The remaining 24 pigs were subsequently randomized to groups subjected to mild hypothermia (HT, n = 12) or non-hypothermia (NT, n = 12). The NT group received the same treatment as the HT group, with the exception of cooling. Randomization was performed using the envelope method.

The animals in the HT group were actively cooled to a target body temperature of 33°C, maintained at this temperature for 12 h, and then actively rewarmed (0.5°C/hour) to 37°C using an intravascular cooling system (Cool Gard XP; Alsius, Los Angeles, CA), in accordance with the landmark study by Bernard et al [4]. During hypothermia induction and maintenance, the pigs received continuous infusion of pancuronium (0.2 mg/kg/h), and their skin was warmed with a quilt to prevent shivering and muscle movement. The target body temperature of 38°C was maintained in SC and NT pigs using an electric fan and ice bags.

Immediately after ROSC, the animals received post-resuscitation intensive care. The animals were injected with 2.5% glucose electrolyte solution and acetated Ringer’s solution to maintain their CVP above 8 mmHg and their MAP above 50 mmHg. In addition, the animals underwent continuous glucose monitoring, and insulin boluses were intermittently administered, targeting serum glucose levels below 150 mg/dL.

### Brain tissue harvesting

Neurologic outcomes were evaluated at 24 hours after ROSC, and the pigs were then euthanized via an overdose of sodium pentobarbital, propofol and potassium chloride. Their brains were immediately removed via craniotomy and divided via a midsagittal cut. The right precentral gyrus of the frontal lobe was dissected, and a portion of the frontal cortex samples (300 to 500 mg) was used to determine brain water content. Another portion of frontal cortex samples was placed on ice and used to observe changes in tissue ultramicrostructure under a transmission electron microscope (TEM). Samples (1 to 2 g) were also collected from the precentral gyrus of the left frontal lobe, from which microvessel segments were rapidly isolated to measure the expression of the TJ proteins occludin, claudin-5, and ZO-1 and the AJ protein VE-cadherin, and additional samples (1 to 2 g) were collected to measure VEGF, Ang-1 and MMP-9 protein expression. The microvessel segment and frontal cortex samples were immediately snap-frozen in liquid nitrogen and stored at -80°C until analysis. These cerebral areas were chosen for analysis because the frontal lobe of the cerebral cortex is closely related to neurological function outcomes.

### Measurement of brain water content

Brain oedema was evaluated via brain cortical water content assessments, which were performed at 24 hours after ROSC. Cortical tissues were immediately divided after craniotomy and weighed to determine their wet weights. These tissues were slowly dried in an oven (90°C) for 72 hours and then reweighed to determine their dry weights [13]. Brain water content (%) was calculated as wet weight−dry weight/wet weight×100% [11,13].

### Measurement of blood–brain barrier permeability

BBB permeability was measured in four pigs randomly selected from each group. The permeability of the BBB was quantitatively evaluated by leakage of Evans blue [11,20,27] in the brain cortex at 24 hours after ROSC. Briefly, Evans blue dye (2%; 5 mL/kg; Sigma, St. Louis, MO, USA) in saline was injected into the left internal jugular vein 2 hours before euthanasia. At 24 hours of ROSC, the pigs were transcardially perfused with saline to remove the intravascular dye, and then the cortex of the right frontal lobe was promptly dissected and rinsed with PBS. The samples were weighed, homogenized in 50% trichloroacetic acid, and centrifuged (10,000 g) for 30 minutes. A 1-mL aliquot of the supernatant was mixed with 3.0 mL of ethanol. The fluorescence of the sample was measured (excitation 620 nm, emission 680 nm) [27] with a spectrophotometer (Hitachi 650–60, Hitachi Ltd., Tokyo, Japan), and the concentration of dye was determined from a standard curve derived from known amounts of dye and its fluorescence intensity. The amount of extravasated dye was expressed as micrograms per gram of brain tissue.

### Transmission electron microscopy

The frontal cortex was cut into sections of approximately 1 mm^3^ thickness on ice. These sections were fixed overnight at 4°C with 2.5% glutaraldehyde and 2.0% paraformaldehyde. Next, the samples were washed three times for 10 min each with 0.1 mol/L phosphate-buffered saline (PBS) and then post-fixed with 1% osmic acid for 2 hours at 4°C. After fixation, the samples were dehydrated in graded acetone and embedded with Epon 812. The semi-thin sections were then cut and double-stained with 2.0% uranyl acetate and 2.0% lead citrate. The BBB ultrastructure was observed blindly and photographed under a TEM (H-7650; Hitachi, Tokyo, Japan).

Three pigs were randomly selected from each group at 24 hours after ROSC, and at least four different electron micrographs representing different areas of cortical microvessels and neuronal mitochondria were selected from each pig. Neuron injury is associated with microvascular damage [28], and neuron cell survival or death depends on critical functions of the mitochondria [29]. To quantitatively assess the ultrastructural changes in mitochondria, we used the morphometric analysis score as described previously [30]. The score includes two components: the matrix and the membrane cristae. The scores from each component were summed. A score of four indicated that the mitochondria were not damaged, whereas a score of zero represented extensive mitochondrial damage.

### Quantitative real-time PCR assay

RT-PCR was used to detect the mRNA expression levels of the TJ-related genes occludin, claudin-5, and ZO-1 and the AJ-related gene VE-cadherin in brain cortical microvessel segments, which were collected as described by Yu et al [22]. Total RNA was extracted from cortical microvessel segments using TRIzol reagent (Invitrogen Corporation, USA) according to the manufacturer’s instructions. First-strand cDNA was synthesized via reverse transcription using a PrimeScript™ 1st Strand cDNA Synthesis Kit (Takara Biomedical Technology, Dalian, China). Real-time PCR was performed using SYBR Green Premix Ex Taq™ (Takara Biomedical Technology, Dalian, China) and Real-Time PCR Detection Systems (Bio-Rad Laboratories, USA). The reaction mixture contained 10 μL of SYBR Green master mix, 1 μL of primers, 1 μL of cDNA, and 8 μL of ddH_2_O. The cycling conditions were as follows: 95°C for 3 min followed by 39 cycles of 95°C for 5 seconds, 56°C for 10 seconds, 72°C for 25 seconds, 65°C for 5 seconds, and 95°C for 50 seconds. Data were analysed using Qbase Plus software. The primer sequences of the specific PCR products were as follows: occludin (forward: 5'-CTTTCTCAGCCAGCGTAT-3' and reverse: 5'-CATCACGATAACGAGCATA-3'), claudin-5 (forward: 5'-CCTTCCTGGACCACAACAT-3') and reverse: 5'-GCACCGAGTCGTACACCTT-3'), ZO-1 (forward: 5'- CAGCCAGTTCATAGCATAG-3' and reverse: 5'-TTCAGGCGAAAGGTAAGG -3'), VE-cadherin (forward: 5'-CTCATCTCGGACAACGG-3' and reverse: 5'-CCATCTCCTCGCACAA-3'), and glyceraldehyde-3-phosphate dehydrogenase (GAPDH) (forward: 5'-TTGTGATGGGCGTGAA-3' and reverse: 5'-TCTGGGTGGCAGTGAT -3'). Gene expression was normalized to GAPDH. Data analysis was performed using the 2^−ΔΔCT^method. All reactions were performed at least three times.

### Western blotting analysis

Western blotting was used to determine the occludin, claudin-5, ZO-1, and VE-cadherin protein expression levels in brain cortical microvessels and the VEGF, Ang-1, and MMP-9 expression levels in brain cortical tissue. Cerebral microvessel segments were obtained from cortical tissue as previously described [22]. These microvessel segment samples and frontal cortical samples were prepared via rapid homogenization in RIPA lysis buffer (Beyotime Inc., Nanjing, China) according to the manufacturer’s instructions. The lysates were centrifuged at 12,000 rpm for 15 min at 4°C to produce whole-cell extracts. Total protein concentrations were determined via BCA assays (Beyotime). Forty micrograms of each sample were added to 12% SDS–polyacrylamide gels and separated via electrophoresis (Mini-Protean III, Bio-Rad, Hercules, CA, USA). The proteins were subsequently transferred onto polyvinylidene difluoride membranes (Millipore, Billerica, MA). The membranes were blocked with PBS containing 5% skimmed milk powder and then incubated overnight at 4°C with primary rabbit antibodies against occludin (diluted 1:1000, Abcam, Cambridge, MA, USA), claudin-5 (diluted 1:300, Biosynthesis Biotechnology, Beijing, China), ZO-1 (diluted 1:200, Abcam), VE-cadherin (diluted 1:1000, Abcam), GAPDH (diluted 1:1000, Cell Signaling Technology, USA), VEGF (diluted 1:2000; Abcam), Ang-1 (diluted 1:500; Abcam), MMP-9 (diluted 1:1000; Abcam) and. β-actin (diluted 1:2000, Biosynthesis Biotechnology, Beijing, China). After washing in PBST buffer (PBS containing 0.05% Tween 20), the membranes were incubated with HRP-conjugated secondary antibodies (1:10,000) for 2 h. GAPDH and β-actin served as a positive control for total protein, and bands were detected using an enhanced chemiluminescence detection system (Tanon, Shanghai, China). The integrated optical densities (IODs) of the protein bands were digitally quantified using Gel-Pro Analyzer version 3.1 (Media Cybernetics, Silver Spring, MD, USA). Occludin, claudin-5, ZO-1, and VE-cadherin protein levels were normalized to GAPDH, and VEGF, Ang-1, and MMP-9 expression levels were normalized to β-actin and presented as a ratio.

### Neurologic outcome assessment

Sedation and analgesia were discontinued at 23 hours after ROSC, and the neurological statuses of all successfully resuscitated animals were evaluated at 24 hours after ROSC using the Overall Performance Category (OPC) score [31,32]. The OPC score scale was based on the Cerebral Performance Category (CPC) score system modified for pigs [32–34]. Briefly, the investigator assessed the responses of the experimental pigs to stimuli such as auditory stimuli, noxious stimuli if unresponsive, and trying to lift the animals, whether the animals could stand, move all limbs, or walk. The OPC score scale is as follows: OPC 1 (normal function: no difficulty standing, walking, drinking, or eating, alert and fully responsive to noxious stimuli); OPC 2 (moderate disability: aware and conscious, standing but an unsteady gait, drinking but not normal eating, and a slower response to noxious stimuli); OPC 3 (severe disability: unable to stand, not drinking or eating, awake but failing to respond normally to noxious stimuli); OPC 4 (vegetative state or coma) and OPC 5 (death).

### Statistical analysis

After confirmation of a normal distribution using the Shapiro-Wilk test, the data were expressed as the means ± sd and were analysed using Student’s t-test for comparisons between two groups or using one-way analysis of variance (ANOVA) followed by Bonferroni’s post hoc test to correct for multiple comparisons. The number of shocks before ROSC, the OPC scores and ultrastructural mitochondria scores, which exhibited a skewed distribution, were expressed as medians (25th and 75^th^ percentiles) and were compared using the Mann-Whitney U test. The ultrastructural changes in TJs and the 24-hour survival rate were compared using Fisher’s exact test. Two-tailed probability values less than 0.05 were considered statistically significant. Analysis was performed using SPSS 21.0 (SPSS, Chicago, IL, USA).

## Results

### Baseline status, resuscitation outcomes, survival and neurologic outcomes

There were no differences in baseline characteristics, including body weight, bladder temperature, ETCO_2_, CVP, MAP, heart rate, cardiac output, arterial oxygen pressure, arterial lactate and base excess, between the groups (*P*>0.05) (Table 1).

**Table 1:** Baseline characteristics.

	SC (n = 8)	HT (n = 12)	NT (n = 12)	*P*
**Weight (kg)**	29.5±2.9	29.3±2.2	30.5±1.8	0.40
**Temperature (bladder, °C)**	38.0±0.1	38.1±0.2	38.0±0.2	0.33
**ETCO_2_ (mmHg)**	42.5±2.1	41.4±1.7	41.7±2.1	0.47
**CVP (mmHg)**	9±1.4	9±1.3	9±1.7	0.99
**MAP (mmHg)**	100.1±2.7	100.8±3.9	100.5±3.5	0.91
**Heart rate (bpm)**	103.6±5.6	101.8±6.3	103.9±7.2	0.69
**CO (L/min)**	4.9±0.6	5.1±0.5	5.1±0.6	0.67
**Arterial PO_2_ (mmHg)**	98.6±1.3	98.8±1.1	98.9±1.2	0.86
**Arterial lactate (mg/L)**	1.3±0.2	1.3±0.3	1.4±0.2	0.86
**BE (mEq/L)**	4.7±0.7	5.2±0.8	4.8±0.7	0.23

Values are presented as means ± sd. HT, mild hypothermia group; NT, non-hypothermia group; SC, surgery control group; bpm, beats per minute; MAP, mean arterial pressure; CVP, central venous pressure; CO, cardiac output; ETCO_2_, end-tidal carbon dioxide; BE, base excess.

**Fig 2:**
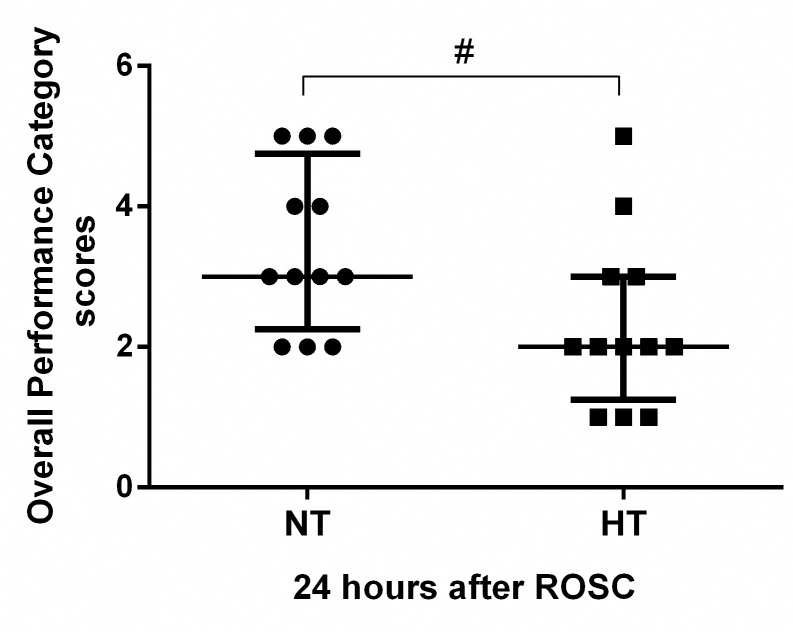
Overall performance category scores. Neurologic outcome was evaluated based on Overall Performance Category scores in the NT (n = 12) and HT (n = 12) groups. A score of 1 represents normal, whereas a score of 5 indicates brain death. Values are given as the median ± interquartile range. ROSC, restoration of spontaneous circulation; NT, non-hypothermia group; HT, mild hypothermia group. ^#^*P*<0.05 versus NT group.

There were no significant differences in the duration of CPR before ROSC (277.9±91.1 seconds versus 258.8±95.8 seconds, *P*>0.05) or the number of shocks before ROSC [2(1.25,3.0) versus 2(1.00,2.75), *P*>0.05)] between the HT (n = 12) and NT (n = 12) groups. Three resuscitated pigs in the NT group (25.0%) and one resuscitated pig in the HT group (8.3%) did not survive for 24 hours, but the difference in survival between the two groups did not reach statistical significance (*P*>0.05). The OPC score of the HT group was significantly better than that of the NT group [2 (1.25, 3) versus 3.0 (2.25, 4.75), *P*<0.05] at 24 hours after ROSC (Fig 2).

**Fig 3:**
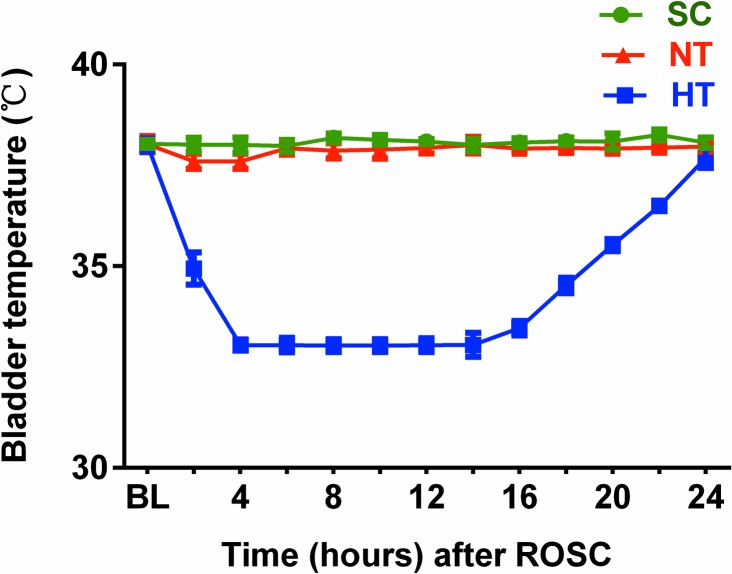
Bladder temperature. BL, baseline; SC, surgery control group; NT, non-hypothermia group; HT, mild hypothermia group.

Compared with the SC and NT groups, the bladder temperature in the HT group decreased to the target temperature (33°C) within 4 h after the start of rapid cooling and remained at this temperature for 12 h, followed by gradual rewarming to 37°C in the HT group (Fig 3).

### Brain oedema after cardiac arrest and resuscitation

**Fig 4:**
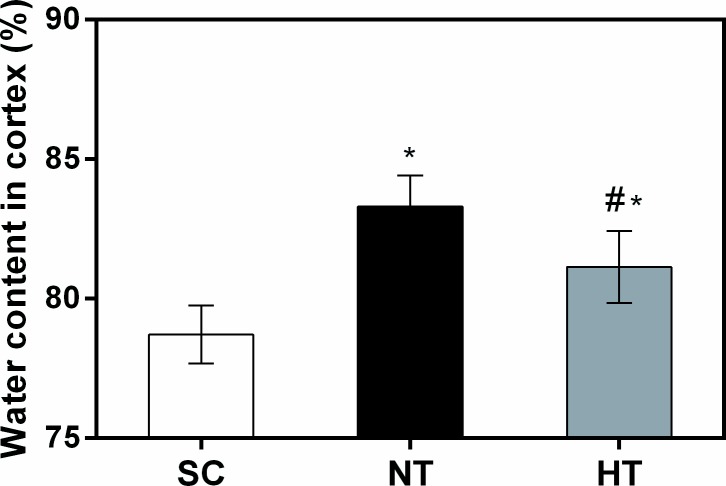
Cortical tissue water content at 24 hours after CA and resuscitation. Brain oedema was calculated as cortical tissue water content in the SC (n = 4), NT (n = 5), and HT (n = 7) groups. Data are presented as means ± sd. SC, surgery control group; NT, non-hypothermia group; HT, mild hypothermia group. *P<0.05 versus SC group; ^#^*P*<0.05 versus NT group.

Cortical tissue water content was 83.30%±1.10% in the NT group and 81.13%±1.29% in the HT group at 24 hours after ROSC (Fig 4). Both values were significantly higher than the water content of the SC group (78.71%±1.04%, both *P*<0.05). However, mild hypothermia treatment significantly decreased cortical tissue water content compared with non-hypothermia treatment (*P* = 0.023).

### Blood–brain barrier permeability induced by cardiac arrest and resuscitation

**Fig 5:**
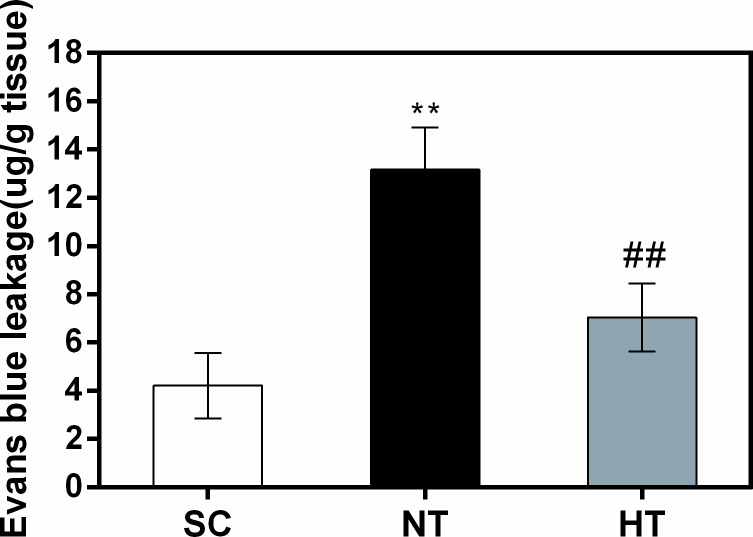
Blood–brain barrier permeability in cortical tissues at 24 hours after CA and resuscitation. Blood–brain barrier permeability was quantitatively evaluated via leakage of Evans blue in the SC (n = 4), NT (n = 4), and HT (n = 4) groups. Data are presented as means ± sd. SC, surgery control group; NT, non-hypothermia group; HT, mild hypothermia group. **P<0.01 versus SC group; ^##^*P*<0.01 versus NT group.

Evans blue binds to serum albumin, which does not cross the BBB under normal physiological conditions. Leakage of albumin into brain tissue occurs only upon BBB permeability breakdown. As shown in Fig 5, compared with the SC group, there was a significant increase in cortical permeability to Evans blue in the NT group (*P*<0.01). However, there was a significant decrease in cortical permeability to Evans blue in the NT group compared with the HT group (*P*<0.01) at 24 hours after ROSC.

### Occludin, claudin-5, ZO-1, and VE-cadherin mRNA and protein expression after cardiac arrest and resuscitation

**Fig 6:**
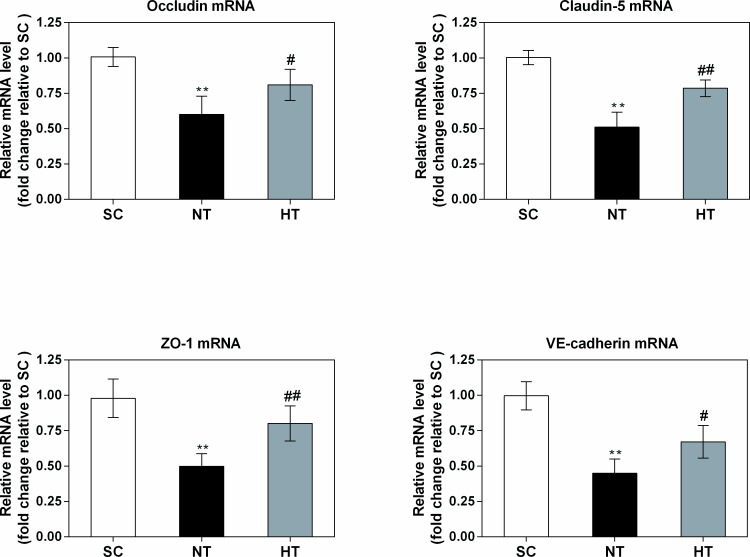
Occludin, claudin-5, ZO-1, and VE-cadherin mRNA expression in cortical tissues at 24 hours after CA. The mRNA levels were quantified via real-time PCR in the SC (n = 4), NT (n = 5), and HT (n = 7) groups, and the data were normalized to GAPDH levels. The fold changes in occludin, claudin-5, ZO-1, and VE-cadherin mRNA expression were calculated relative to the expression levels in the SC group, and the results are presented as the mean fold changes ± sd relative to the SC group. SC, surgery control group; NT, non-hypothermia group; HT, mild hypothermia group. *P<0.05, **P<0.01 versus SC group; ^#^*P*<0.05, ^##^*P*<0.01 versus NT group.

RT-PCR and western blotting were performed to determine the effects of mild hypothermia on occludin, claudin-5, ZO-1, and VE-cadherin expression in cortical tissue 24 hours after ROSC. As shown in Fig 6, we observed significant (all *P<*0.01) decreases in the mRNA expression levels of occludin (0.60±0.13-fold), claudin-5 (0.51±0.10-fold), ZO-1 (0.50±0.09-fold) and VE-cadherin (0.45±0.10-fold) in the NT group relative to the SC group at 24 hours after ROSC. Compared to the NT group, we observed significantly (all *P*<0.05) smaller decreases in the mRNA expression levels of these proteins in the cortical tissue of the HT group at 24 hours after ROSC (occludin: 0.81±0.11-fold relative to the SC group; claudin-5: 0.79±0.06-fold relative to the SC group; ZO-1: 0.80±0.12-fold relative to the SC group; and VE-cadherin: 0.67±0.12-fold relative to the SC group).

**Fig 7:**
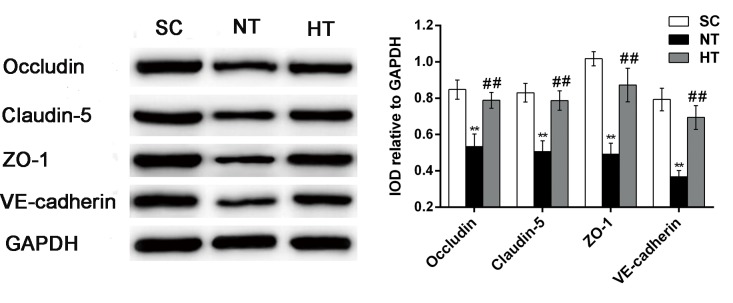
Occludin, claudin-5, ZO-1, and VE-cadherin protein expression in cortical tissues at 24 hours after CA. Western blotting (left) was used to measure occludin, claudin-5, ZO-1, and VE-cadherin protein levels in the SC (n = 4), NT (n = 5), and HT (n = 7) groups. The IOD of each band was measured using Gel-pro Analyzer software. Occludin, claudin-5, ZO-1, and VE-cadherin levels were normalized to GAPDH levels, and the results are presented as the mean ± sd. SC, surgery control group; NT, non-hypothermia group; HT, mild hypothermia group. **P<0.01 versus SC group; ^##^*P*<0.01 versus NT group.

As shown in Fig 7, the cortical protein expression levels of occludin, claudin-5, ZO-1 and VE-cadherin were significantly decreased in the NT group at 24 hours post-ROSC compared with the SC group (all *P<*0.01). However, compared with the NT group, significant attenuation of these reductions in the protein levels of occludin, claudin-5, ZO-1 and VE-cadherin were observed in the HT group (all *P*<0.01).

### Vascular endothelial growth factor, angiogenin-1 and matrix metalloproteinase-9 expression after cardiac arrest and resuscitation

**Fig 8:**
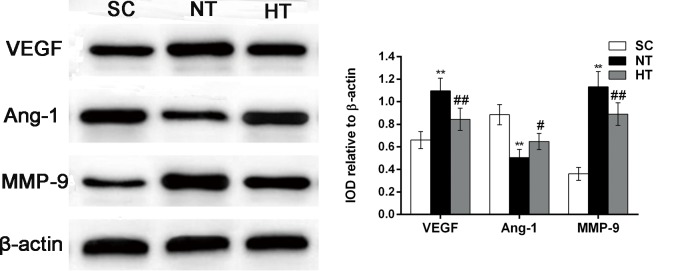
VEGF, Ang-1 and MMP-9 protein expression in cortical tissues at 24 hours after CA. The expression of VEGF, Ang-1 and MMP-9 was measured by western blotting (left) in the SC (n = 4), NT (n = 5), and HT (n = 7) groups. VEGF, Ang-1 and MMP-9 levels were normalized to β-actin and are presented as the mean ± sd. SC, surgery control group; NT, non-hypothermia group, HT, mild hypothermia group; VEGF, vascular endothelial growth factor; Ang-1, angiogenin-1; MMP-9, matrix metalloproteinase-9. *P<0.05, **P<0.01 versus SC group; ^#^*P*<0.05, ^##^*P*<0.01 versus NT group.

As mentioned above, VEGF, Ang-1 and MMP-9 play central roles in maintaining TJ and AJ barrier integrity. To elucidate the molecular mechanisms underlying CA- and resuscitation-induced TJ and AJ alterations, we studied VEGF, Ang-1 and MMP-9 protein expression in the brain cortex at 24 hours after ROSC. As shown in Fig 8, compared with the SC group, we observed a significant increase in VEGF protein expression in the NT group (*P*<0.01). However, we observed a significant decrease in VEGF protein expression in the HT group (*P*<0.01, compared with the NT group). A decrease in Ang-1 protein expression was observed in the NT group compared with the SC group (*P*<0.01). In contrast, this decrease in Ang-1 levels was attenuated in the HT group (*P*<0.05, compared with the NT group). Significant increases in MMP-9 expression levels in the cortex were observed in the NT group compared with the SC group (*P*<0.01). However, these increases in MMP-9 levels were significantly attenuated in the HT group (*P*<0.01, compared with the NT group).

### Ultrastructural changes in cortical tissues after cardiac arrest and resuscitation

**Fig 9:**
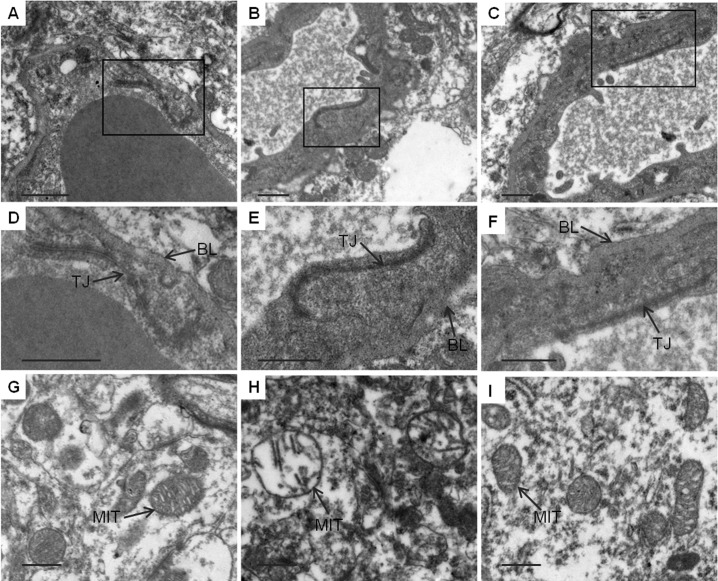
TEM observation of ultrastructural alterations in cortical tissues 24 hours after CA and resuscitation. Normal TJs, basement membranes and neuronal mitochondria were observed in the SC group (A, D, G). In the NT group, the TJs were obscure, and the basement membranes were disrupted; moreover, neuronal mitochondria were markedly swollen, with disrupted cristae (B, E, H). In the HT group, the TJs and basement membrane were continuous, and neuronal mitochondria were slightly damaged, with normal cristae (C, F, I). SC, surgery control group; NT, non-hypothermia group; HT, mild hypothermia group; TJ, tight junction; BL, basement membrane; MIT, mitochondria. Scale bars = 2 μm.

Changes in TJ barrier and neuronal mitochondria integrity after CA and resuscitation were investigated by TEM. As shown in Fig 9A, 9D and 9G, the TJs between endothelial cells remained intact in the SC group and appeared as a series of electron-dense zones resting against the plasma membranes of adjacent endothelial cells and sealing intercellular clefts in normal brain microvessels. The basement membrane was also continuous and integrated, and normal neuronal mitochondria structure was observed. By contrast, among the fifteen TJs and adjacent basement membrane observed in the NT group (Fig 9B and 9E), twelve were obscure, and the adjacent basement membrane had collapsed at 24 hours after ROSC. However, as shown in Fig 9C and 9F, mild hypothermia attenuated the TJ and basement membrane breakdown, and among sixteen TJs and adjacent basement membranes observed, five were disrupted. The difference between the NT group and the HT group was statistically significant (12/15 vs 5/16, *P*<0.05). As shown in Fig 9H, most of the neuronal mitochondria were severely swollen, with no homogenous density in the matrix and disrupted membrane cristae at 24 hours after ROSC in the NT group. However, the ultrastructural damage of neuronal mitochondria in the cerebral cortex was reduced in the HT group (Fig 9I) compared to the NT group with a significantly elevated score [3 (2.25, 3) vs 2 (1.25, 2.0), *P*<0.01].

## Discussion

Neurological dysfunction resulting from CA is closely associated with high post-resuscitative mortality rates and poor quality of life [35,36]. We employed a pig model and demonstrated that global cerebral ischaemia following CA and CPR resulted in BBB disruption and oedema in the cerebral cortex at 24 hours after ROSC. More importantly, we showed that whole-body mild hypothermia attenuated cortical oedema and BBB disruption at 24 hours post-ROSC. This conclusion was based on our findings that mild hypothermia (1) decreased cortical water content and BBB permeability; (2) upregulated cortical protein and mRNA expression of the TJ-related occludin claudin-5, ZO-1 and the AJ-related VE-cadherin; (3) increased cortical Ang-1 protein expression and suppressed cortical VEGF and MMP-9 protein expression. Furthermore, at 24 hours after ROSC, whole-body mild hypothermia improved neurologic outcomes, a finding consistent with those of previous reports involving animals and humans [4–6].

Brain oedema, defined as a local or diffuse increase in the volume of fluid in the brain parenchyma, can be divided into cytotoxic oedema and vasogenic oedema. Cerebral ischaemia primarily results in cytotoxic oedema, and vasogenic oedema plays a key role in brain oedema development after reperfusion [3]. CA and resuscitation cause systemic ischaemia-reperfusion injury, also referred to as postcardiac arrest syndrome [36], which can result in cytotoxicity and vasogenic oedema [3]. Diffuse brain oedema can exacerbate brain injury in CA patients and has been shown to be predictive of poor neurologic outcome [2]. Xiao et al. [1] demonstrated that CA and resuscitation resulted in brain oedema development at one hour after ROSC in an 8-minute asphyxiation model of CA in rats. In the present study, we employed a pig model and demonstrated that global cerebral ischaemia following CA and CPR resulted in BBB disruption and oedema in the cerebral cortex at 24 hours after ROSC. Furthermore, we showed that whole-body mild hypothermia attenuated cortical oedema and BBB permeability. The potential relationship among these results is unclear. However, the beneficial effect of mild hypothermia likely starts with restoration of BBB permeability, followed by alleviation of brain oedema at 24 hours post-ROSC.

The BBB shields the brain parenchyma from plasma components, including ions and macromolecules. BBB breakdown, resulting in permeability to plasma proteins, leads to vasogenic oedema, as clearly established in experimental models and in humans suffering from various types of acute brain injury, such as infarcts, tumours, trauma, haemorrhages, infections, inflammation, and epilepsy [37]. Previous studies had shown that CA and resuscitation could induce BBB breakdown [11,12,14,38]. For example, Pluta et al. [12] reported that the BBB opened in a biphasic manner in a rat CA model. The first phase occurred immediately after CA and lasted for 1 hour; the second phase occurred between 6 and 24 hours after ROSC. Another study of pigs subjected to CA induced by 9 minutes of asphyxia revealed a significant increase in BBB permeability after 2 hours of reperfusion [38]. Miclescu et al. [14] demonstrated that BBB disruption begins during the initial phase of untreated CA and progresses in a time-dependent manner. The discrepancy between these results and those of the present study may be related to differences in the species tested, the methods of assessing BBB function, and the time points of investigation. Previous studies have shown that hypothermia has a protective effect on the integrity of the BBB in global ischaemia. Baumann et al. [39] reported that hypothermia immediately following cerebral artery occlusion in rats was associated with reduced breakdown of the microvascular basal lamina. Another study [40] showed that six hours of hypothermia treatment prevented delayed BBB opening in a rat model of global ischaemia, and hypothermia was also effective when initiated at 1 h but not 2 h following ischaemia. Two animal experiments employing a bilateral common carotid artery occlusion ischaemia-reperfusion procedure and treatments have been performed. However, the pathophysiological mechanisms of brain injury in this ischaemia-reperfusion model are not fully identical to those of brain injury from CA and resuscitation. In the present study, using a CA pig model and transmission electron microscopy, we observed ultrastructural changes in the TJs between cerebral microvascular endothelial cells and that the adjacent basement membranes had collapsed at 24 hours following ROSC. Furthermore, the neuronal mitochondria were severely swollen. Additionally, we showed for the first time that mild hypothermia attenuated CA- and resuscitation-induced TJ ultrastructure breakdown in a swine model.

BBB disruption is closely related to structural and functional alterations in the TJs and AJs between adjacent microvascular endothelial cells. TJs are recognized as the primary paracellular barrier, and AJs play key roles in TJ localization and stabilization [41]. To elucidate the mechanisms underlying BBB breakdown after CA and resuscitation, we investigated the potential changes in TJ and AJ gene and protein expression at 24 hours after ROSC. The TJ-related proteins claudin-5, occludin, and ZO-1 are thought to be sensitive indicators of normal BBB function and BBB perturbation [23]. The transmembrane proteins claudin-5 and occludin are recognized to be directly involved in regulating of BBB integrity and function [42,43], and occludin has been shown to be directly responsible for forming TJ strands. BBB disruption is typically accompanied by the absence of occludin expression [44]. Claudin-5 is a key cell adhesion molecule in the TJs of brain endothelial cells, and claudin-5 knockout mice are characterized by size-selective BBB defects [45]. ZO-1 serves as a recognition protein for TJ placement and as a signal transduction support protein. This protein plays an important role in connecting transmembrane proteins with cytoskeletal proteins [9,18,46]. Loss of ZO-1 can result in TJ disorganization [16]. VE-cadherin, an important determinant of microvascular integrity both in vivo and in vitro [47], mediates cell adhesion in a Ca^2+^-dependent manner at cell junctions and decreases cell permeability [41]. A previous study [1] observed no hypothermia-induced changes in occludin expression during the early phase of ROSC in rats subjected to CA induced via 8 min of asphyxia; however, that study tested the level of occludin expression after one hour of ROSC, and the method of induction mild hypothermia before CA is not clinically accepted. VF is known to be the leading cause of CA in adults, and the metabolic and cardiovascular functions of pigs are very similar to those of humans; thus, we used a VF pig model to elucidate the specific roles of the three abovementioned TJ proteins as well as VE-cadherin in CA- and CPR-induced BBB breakdown. We observed that the mRNA and protein expression levels of these three TJ-related proteins as well as VE-cadherin were significantly decreased at 24 hours after ROSC, suggesting that TJ and AJ weakening led to BBB opening. More importantly, we observed that mild hypothermia treatment significantly attenuated the CA- and resuscitation-induced reductions in the protein and mRNA expression of occludin, claudin-5, ZO-1, and VE-cadherin. These findings suggest that mild hypothermia protects against BBB disruption, which might be associated with the attenuation of TJ and AJ breakdown at 24 hours post-ROSC.

The disruption of BBB under ischaemic conditions is multifactorial and may involve factors such as enhanced production of inflammatory cytokines, excessive oxidative stress, and upregulation of VEGF [17]. Therapeutic hypothermia can decrease the expression of inflammatory mediators and free radical generation [5,7,30]. We measured cortical MMP-9, VEGF and Ang-1 expression levels at 24 hours following ROSC to investigate the possible molecular mechanisms underlying the neuroprotective effects of mild hypothermia against CA- and CPR-induced TJ and AJ disruption. MMP-9 has been confirmed as a mediator of brain oedema in both cerebral ischaemia and CPR studies [3,5,11,17]. MMP-9 degrades the extracellular matrix of the basement membrane as well as TJ proteins, thereby disrupting BBB integrity [21,48]. VEGF is another factor that effectively increases microvascular permeability to blood plasma proteins within a few minutes after exposure [49]. VEGF has been reported to increase BBB permeability by downregulating TJ protein expression [50–52]. For example, Pichiule et al. [53] reported that VEGF levels were significantly increased in rat brain cortical, hippocampal and brainstem tissues at 24 and 48 hours after CA and resuscitation. Ang-1 protects against vascular leakage in adults, and prevents VEGF-induced increases in TJ permeability at the BBB by decreasing MMP-9 activity [20]. A recent study suggested that Ang-1 decreases BBB permeability and brain infarct volumes in middle cerebral artery occlusion and reperfusion in rats and that these effects are related to the ability of Ang-1 to upregulate ZO-1, occludin, and VE-cadherin [22]. Another recent study [11] showed significant increases in MMP-9 and VEGF expression levels and decreases in Ang-1 expression levels in the cortex and hippocampus at 24 hours after CA and resuscitation, indicating that these factors are involved in BBB permeability in the rat brain. Based on these findings, we studied the effects of mild hypothermia on the expression of MMP-9, VEGF and Ang-1 in the cerebral cortex at 24 hours after ROSC. We observed decreases in MMP-9 and VEGF protein expression and increases in Ang-1 expression in the HT group compared with the NT group. Thus, we speculate that mild hypothermia has neuroprotective effects against TJ and AJ degradation induced by CA and resuscitation, at least partially by suppressing VEGF and MMP-9 protein expression and increasing Ang-1 protein expression at 24 hours after ROSC.

There are some limitations to this study. First, we focused on CA- and CPR-induced brain oedema only after rewarming (at 24 hours after ROSC), but the changes during the brain perfusion period and during mild hypothermia were not investigated. In addition, we did not discriminate cytotoxic and vasogenic oedema. These changes will be examined in real time by magnetic resonance imaging in future studies. Second, we did not assess histopathology and cellular findings in the brain cortex at 24 hours after ROSC, which would be very useful and might help explain the behavioural findings [54]. Third, we failed to find a significant effect of mild hypothermia on the survival rate, which may be due to insufficient sample size, and the animals were killed immediately at the end of the study, resulting in a lack of observation of the sufficient treatment duration for histological and behavioural protection [54]. Fourth, this experiment involved only young healthy animals. However, most patients who experience CA in clinical practice are old and suffer from chronic diseases. Fifth, we examined only the cerebral cortex and thus did not evaluate other areas vulnerable to the effects of global ischaemia, such as the hippocampus [13]. Sixth, inflammatory mediators, free radicals, or microRNAs as contributing factors to TJ and AJ disruption were not measured in the present study [23]. Finally, due to the use of an intravascular cooling method, the investigator could not be blinded throughout the experiment; however, a person who was blinded to the treatment conditions analyzed the serum and tissue samples and performed all the neurologic evaluations.

## Conclusions

Mild hypothermia protects against early brain oedema and BBB disruption and improves neurologic outcome after CA and resuscitation in pigs. The mechanism underlying these protective effects likely involves attenuation of the breakdown of TJ proteins (occludin, claudin-5, and ZO-1) and AJ protein (VE-cadherin), which may, at least in part, be related to modulation of the expression of MMP-9, VEGF, and Ang-1.

## Supporting information

S1 Data Raw data. (RAR)
